# What guidance is available for researchers conducting overviews of reviews of healthcare interventions? A scoping review and qualitative metasummary

**DOI:** 10.1186/s13643-016-0367-5

**Published:** 2016-11-14

**Authors:** Michelle Pollock, Ricardo M. Fernandes, Lorne A. Becker, Robin Featherstone, Lisa Hartling

**Affiliations:** 1 Alberta Research Centre for Health Evidence, Department of Pediatrics, University of Alberta, Edmonton, AB Canada; 2 Clinical Pharmacology Unit, Instituto de Medicina Molecular, University of Lisbon, Lisbon, Portugal; 3 Department of Pediatrics, Santa Maria Hospital, Lisbon, Portugal; 4 Department of Family Medicine, SUNY Upstate Medical University, Syracuse, NY USA

**Keywords:** Overview of reviews, Umbrella review, Evidence synthesis, Knowledge synthesis, Evidence-based medicine, Evidence-based practice, Review methods, Systematic reviews, Scoping review, Metasummary

## Abstract

**Background:**

Overviews of reviews (overviews) compile data from multiple systematic reviews to provide a single synthesis of relevant evidence for decision-making. Despite their increasing popularity, there is limited methodological guidance available for researchers wishing to conduct overviews. The objective of this scoping review is to identify and collate all published and unpublished documents containing guidance for conducting overviews examining the efficacy, effectiveness, and/or safety of healthcare interventions. Our aims were to provide a map of existing guidance documents; identify similarities, differences, and gaps in the guidance contained within these documents; and identify common challenges involved in conducting overviews.

**Methods:**

We conducted an iterative and extensive search to ensure breadth and comprehensiveness of coverage. The search involved reference tracking, database and web searches (MEDLINE, EMBASE, DARE, Scopus, Cochrane Methods Studies Database, Google Scholar), handsearching of websites and conference proceedings, and contacting overview producers. Relevant guidance statements and challenges encountered were extracted, edited, grouped, abstracted, and presented using a qualitative metasummary approach.

**Results:**

We identified 52 guidance documents produced by 19 research groups. Relatively consistent guidance was available for the first stages of the overview process (deciding when and why to conduct an overview, specifying the scope, and searching for and including systematic reviews). In contrast, there was limited or conflicting guidance for the latter stages of the overview process (quality assessment of systematic reviews and their primary studies, collecting and analyzing data, and assessing quality of evidence), and many of the challenges identified were also related to these stages. An additional, overarching challenge identified was that overviews are limited by the methods, reporting, and coverage of their included systematic reviews.

**Conclusions:**

This compilation of methodological guidance for conducting overviews of healthcare interventions will facilitate the production of future overviews and can help authors address key challenges they are likely to encounter. The results of this project have been used to identify areas where future methodological research is required to generate empirical evidence for overview methods. Additionally, these results have been used to update the chapter on overviews in the next edition of the *Cochrane Handbook for Systematic Reviews of Interventions*.

**Electronic supplementary material:**

The online version of this article (doi:10.1186/s13643-016-0367-5) contains supplementary material, which is available to authorized users.

## Background

Systematic reviews (SRs) combine the results of multiple similar primary studies to answer a specific clinical question [1]. With the exponential increase in the number of published SRs [2], overviews of reviews (overviews) have emerged as a logical solution to help manage this information overload. The purpose of overviews is to integrate information from multiple related SRs to provide a comprehensive synthesis of all SR evidence related to a specific clinical question [3]. They are designed to be accessible, user-friendly documents that are typically broader in scope than any individual SR. Overviews are often conducted to address questions related to the efficacy, effectiveness, and/or safety of healthcare interventions—for example, examining multiple interventions for the prevention or treatment of a specific health condition [3]. Table 1 describes the key characteristics of overviews.

**Table 1:** Key characteristics of overviews of reviews

1) Overviews should contain a clearly formulated objective designed to answer a specific clinical research question, typically about a healthcare intervention.
2) Overviews should intend to search for and include only systematic reviews (with or without meta-analyses).
3) Overviews should use explicit and reproducible methods to identify multiple systematic reviews that meet their inclusion criteria and to assess the methodological quality of these systematic reviews.
4) Overviews should intend to collect, analyze, and present the descriptive characteristics of their included systematic reviews (and their primary studies) and the quantitative outcome data contained within the systematic reviews.

Modified from Becker and Oxman and Hartling et al. [3, 4]

Given their objective to synthesize extensive data in a user-friendly format, overviews have been gaining momentum as a valuable knowledge synthesis product to facilitate the uptake and application of knowledge by decision-makers. Thus, the number of published overviews has been steadily increasing in recent years [4–6]. This increase is at least partially due to the pioneering efforts of The Cochrane Collaboration, an international organization widely recognized as producing high-quality SRs of health evidence [7]. In 2004, the Comparing Multiple Interventions Methods Group (originally called the Umbrella Reviews Methods Group) was established to develop general guidance for conducting overviews [8]. This preliminary guidance was first published as a chapter in the *Cochrane Handbook for Systematic Reviews of Interventions (Cochrane Handbook)* in 2008 [3], and the first overview was published in the Cochrane Database of Systematic Reviews (CDSR) in 2009 [9]. Today, Cochrane authors can publish overviews in the CDSR with a label that allows readers to distinguish them from standard SRs. Other research groups and organizations around the world have also adopted this research design as a valuable knowledge synthesis product [10, 11].

Overview methods evolved from SR methods for which there are well-established standards of conduct to ensure rigor, validity, and reliability of results [12]. Overviews therefore aim to use explicit, reproducible, and systematic methods to search for, identify, and extract outcome data from SRs. However, since the unit of searching, inclusion, and data extraction is the SR, overview authors often encounter unique methodological challenges for which there are no obvious solutions or clear guidance. As a result, current practice when conducting overviews is driven largely by personal experience and trial and error, and published overviews show considerable variation in their methods and reporting [4–6]. In recent years, a number of overview authors have recognized the methodological challenges inherent in conducting overviews and expressed a need for comprehensive, up-to-date guidance for overviews [4, 5, 13].

The purpose of this scoping review was to identify and summarize all documents containing methodological guidance for conducting overviews examining the efficacy, effectiveness, and/or safety of healthcare interventions. The aims were as follows: (1) locate, access, compile, and create a map of documents that provide explicit methodological guidance for conducting overviews; (2) identify and describe areas where guidance for conducting overviews is clear and consistent, as well as areas where guidance is conflicting or missing; and (3) document common challenges involved in conducting overviews and determine whether existing guidance can help researchers overcome these challenges. We then used the results of this scoping review to update the chapter on overview methods appearing in the *Cochrane Handbook*.

## Methods

This scoping review adhered to the methods established by Arksey and O’Malley [14] and expanded upon by Levac et al. [15].

### Eligibility criteria

To be included in the scoping review, documents had to meet one of two criteria: (1) provide explicit guidance for conducting overviews of healthcare interventions, defined as any guidance related to either the context or the process of conducting an overview or (2) describe an author team’s experience conducting one or more overviews of healthcare interventions. When selecting documents for inclusion, we used the definition of overviews provided in Table 1. We included guidance that applied to overviews examining the efficacy, effectiveness, and/or safety of healthcare interventions and excluded guidance that applied to other types of overviews (e.g., diagnostic test accuracy, prognostic, and qualitative overviews). We included documents produced in any language, year, or format.

### Search methods for identification of documents

Our scoping review aimed to identify and include a wide range of document types, including unpublished documents such as internal documents, training manuals, and conference proceedings. We therefore conducted an iterative and extensive search to ensure breadth and comprehensiveness of coverage [14–17]. The search was conducted between January and March 2014 and involved reference tracking, database and web searches, handsearching of websites and conference proceedings, and contacting producers of overviews.

Our iterative reference tracking (“snowballing”) search [16, 17] was conducted by a research librarian (RF). The reference tracking search used a total of 30 target articles about overviews that were identified by the study authors prior to the start of the search and as the search progressed. For each target article, we searched for “citing” references using Google Scholar, “cited” references using Scopus and reference lists, and “similar articles” using PubMed. Database and web searches were conducted to supplement and enhance our reference tracking search (RF). We first updated the database searches reported in Hartling et al. [4]. This involved searching MEDLINE (via Ovid), EMBASE (via Ovid), DARE (via Cochrane Library), and Scopus, for articles published between January 2010 and December 2013. We then augmented this search with two additional databases (MEDLINE via Web of Science, and Cochrane Methods Studies Database via Ovid) and one additional web search engine (Google Scholar). Relevant articles identified by the database and web searches were fed back into the reference tracking search and used as target articles to help locate additional relevant articles.

A number of additional sources were searched in an attempt to locate all unpublished and internal guidance documents (MP, RF). We handsearched the websites of 26 organizations that we knew had published at least one overview, and the conference proceedings (2000–2013) of three conferences: the International Cochrane Colloquium, Health Technology Assessment International, and the Canadian Agency for Drugs and Technologies in Health Symposium. Additionally, we contacted overview producers to ask if they had followed any specific guidance when conducting their overview(s): this involved contacting 20 Managing Editors of Cochrane Review Groups and Fields who oversaw the preparation of a combined 64 overviews published in the CDSR and *Evidence-Based Child Health: A Cochrane Review Journal*, and 110 authors who published a combined 148 overviews in journals other than the CDSR (lists of overviews obtained from [4] and [5]). We had satisfactory response rates (57% for authors of conference proceedings, 96% for Managing Editors of Cochrane Review Groups and Fields, and 55% for overview authors).

We updated select components of the search in November 2015. To ensure we continued to capture relevant documents published after our search dates, we used article alerts from MEDLINE (via Web of Science) and Google Scholar to monitor new articles between January 2014 and November 2015. Additionally, we searched conference proceedings for 2014 and 2015 and contacted an additional five Managing Editors of Cochrane Review Groups who oversaw the preparation of five overviews published in the CDSR in 2014 and 2015.

Finally, we handsearched the reference lists of the 52 guidance documents included in this scoping review. Due to the variability in terminology used to refer to overviews [4], we searched for and included terms such as “overview,” “overview of reviews,” “overview of systematic reviews,” “umbrella review,” “systematic review of systematic reviews,” and “metareview”. See Additional file 1 for complete search strategies.

### Selection of documents

All titles and abstracts were independently screened by one reviewer (MP) and one research assistant. We kept those documents that were not overviews but that met the broad definition of “being about overviews” or “discussing some aspect of overviews.” We then retrieved the full text of all potentially relevant titles and abstracts. Full-text articles were assessed for inclusion by two independent reviewers (MP, LH) using the previously described eligibility criteria, with discrepancies resolved through discussion.

### Data extraction and analysis

Relevant text contained within each included document was extracted and analyzed using a qualitative metasummary approach, which is an iterative, quantitatively oriented method of data analysis that involves aggregating textual data to identify and expose patterns of findings across groups of related documents [18, 19]. This involved extracting, editing, grouping, abstracting, and presenting findings (this work was completed using Microsoft Word and Excel). All data collection and analysis was conducted by one reviewer (MP) and checked for accuracy by a second reviewer (LH), with disagreements resolved through discussion. The qualitative metasummary process is described below.

First, we clearly specified the text eligible and not eligible for extraction using the criteria presented in Additional file 2 [18]. For documents that *provided explicit guidance for conducting overviews of healthcare interventions*, we extracted text that provided guidance on how to conduct any part of an overview and text that described challenges involved when conducting overviews. For documents that *described an author team*’*s experience conducting one or more overviews of healthcare interventions*, we extracted only text that described challenges author teams encountered. We then separated guidance statements and challenges from all other text in the documents, and edited the guidance and challenges to ensure that they were presented in a way that was accessible to readers while preserving their underlying content and meaning [18]. Guidance statements and challenges were then separated from each other and grouped, abstracted, and presented in parallel.

For both guidance statements and challenges, we used a two-stage approach to group similar findings together. First, we grouped all documents produced by the same research group to avoid giving extra weight to statements that were included in multiple documents produced by the same research group [18]. Within each of these groupings, we further edited the findings to eliminate redundancies and duplicate text while leaving the meaning of the statements unchanged. Second, we grouped statements across research groups by stage of the overview process to ensure that all statements related to the same stage of the overview process appeared in the same place [18]. The stages of the overview process included the following: deciding when and why to conduct an overview, specifying the scope, searching for and including SRs, quality assessment of SRs and their primary studies, collecting and analyzing data, grading quality of evidence, and drawing conclusions. These stages were identified iteratively: they were selected in advance using the stages presented in the *Cochrane Handbook* [12] and modified as needed to accommodate the specific guidance and challenges identified.

We then abstracted findings to summarize the content of each group of topically related guidance statements and challenges [18, 19]. For each stage of the overview process, we reworked our lists of guidance statements and challenges until we developed a new list of abstracted statements that captured the overall meaning of the original statements. This was done by eliminating redundancies, refining statements to ensure they were inclusive of the ideas presented by each research group, preserving ambiguity and contradictions, and ensuring clarity and accessibility.

Lastly, we provided a narrative summary of the abstracted guidance statements followed by a narrative summary of the abstracted challenges. For guidance statements only, we also calculated frequency and intensity effect sizes. These were used to extract more meaning from the narrative summaries by numerically describing the magnitude of the abstracted findings [18, 19]. Frequency effect sizes were calculated by dividing the number of research groups contributing guidance on a topic area by the total number of research groups. Intensity effect sizes were calculated by dividing the number of topic areas addressed by each research group by the total number of topic areas.

## Results

### Results of the search

**Fig. 1:**
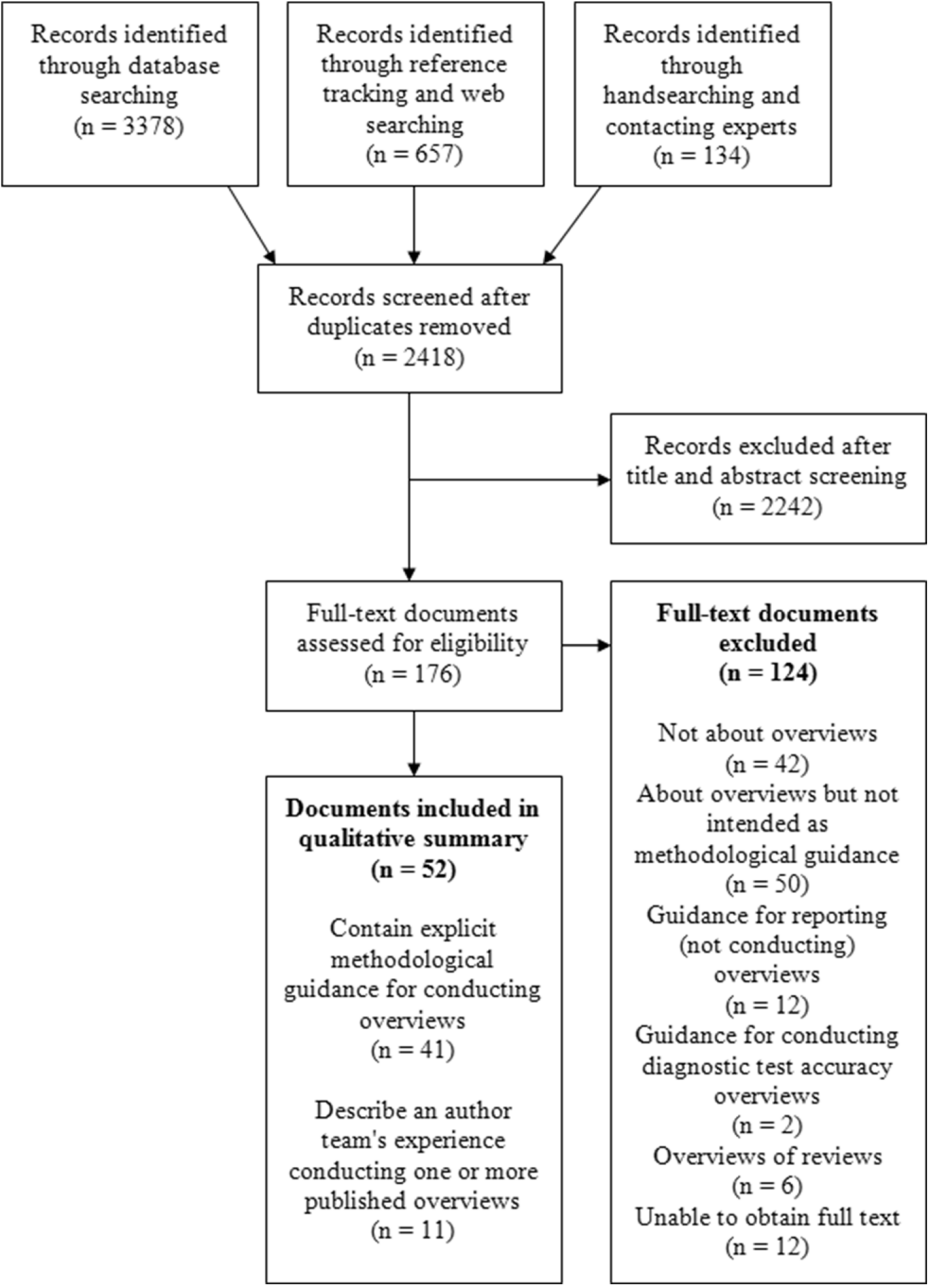
Flow diagram of documents through the scoping review

The literature search retrieved 2418 unique references. One hundred seventy-six references were identified as potentially eligible, and the full-text articles were assessed for inclusion. Of these, 124 documents were excluded (list available upon request). Fifty-two documents produced by 19 research groups were included; these documents are listed in Additional file 3 and are labeled “A1,” “A2,”…, “A52” in the text below. Figure 1 contains a flow diagram of documents through the review process. As anticipated, published articles that could be located through database searching comprised a minority (29%) of included documents; the majority (71%) were unpublished documents identified through other searching methods.

### Summary of included guidance documents

Table 2 summarizes the characteristics of the included guidance documents and presents abbreviations for research groups that will be used throughout the remainder of the results.

**Table 2:** Characteristics of included guidance documents (52 documents produced by 19 research groups)

	Documents that contain explicit methodological guidance for conducting overviews (41 documents produced by 12 research groups)			Documents that describe an author team’s experience conducting one or more published overviews (11 documents produced by 9 research groups)		
Research group	Number of documents (Additional file 3 references)	Years documents were produced	Document formats	Number of documents (Additional file 3 references)	Years documents were produced	Document formats
Cochrane Child Health Field (CHF)	11^a^ (A1–A11)	2010–2015	6 oral presentations^a^, 2 internal documents, 2 posters, 1 journal article	2 (A42, A43)	2011–2013	1 journal article, 1 poster
Cochrane Comparing Multiple Interventions Methods Group (CMIMG)	18^a^ (A1, A6, A7, A12–A26)	2008–2015	10 oral presentations^a^, 5 internal documents, 1 journal article, 1 book chapter, 1 website	–	–	–
Cochrane Consumers and Communication Review Group (CCRG)	–	–	–	1 (A44)	2009	1 journal article
Cochrane Effective Practice and Organization of Care Review Group (EPOC)	1 (A27)	2011	1 oral presentation	2 (A45, A46)	2011–2015	1 oral presentation, 1 poster
Cochrane Musculoskeletal Review Group (CM)	–	–	–	1 (A47)	2010	1 poster
Cochrane Public Health Group (CPHG)	1 (A28)	2014	1 journal article	–	–	–
Cochrane Stroke Group (CS)	–	–	–	1 (A48)	2015	1 oral presentation
Duke University (DukeU)	1 (A29)	2012	1 journal article	–	–	–
Dutch Cochrane Centre (DCC)	–	–	–	1 (A49)	2009	1 poster
Evidence for Policy and Practice Information and Co-ordinating Centre (EPPI)	2 (A30, A31)	2015	1 journal article, 1 oral presentation	–	–	–
Joanna Briggs Institute Umbrella Reviews Methodology Group (JBI)	4 (A32–A35)	2007–2015	2 internal documents, 1 journal article, 1 book chapter	–	–	–
Ludwig Boltzmann Institute for Health Technology Assessment (LBI)	–	–	–	1 (A50)	2015	1 journal article
Norwegian Knowledge Centre for the Health Services (NOKC)	1 (A36)	2013	1 book chapter	–	–	–
Pontifical Xavierian University (PXU)	–	–	–	1 (A51)	2011	1 poster
Trinity College Dublin (TCD)	1 (A37)	2011	1 journal article	–	–	–
University of Birmingham (UBirm)	1 (A38)	2012	1 journal article	–	–	–
University of Dundee (UDun)	–	–	–	1 (A52)	2004	1 journal article
Western Journal of Nursing Research (WJNR)	1 (A39)	2014	1 editorial	–	–	–
Witten/Herdecke University (WHU)	2 (A40, A41)	2014	2 journal articles	–	–	–

^a^Three documents are counted twice because they were produced by authors affiliated with both of these groups (Additional file 3, references ﻿A1, ﻿A6, and ﻿A7). For these three documents, guidance presented by DC, LAB, and RMF was extracted into the CMIMG category, and guidance presented by DT, LH, and MF was extracted into the CHF category

Of the 52 included documents, 41 *provided explicit methodological guidance for conducting overviews of healthcare interventions* (Additional file 3, references A1–A41). These documents were produced between 2007 and 2015 by 12 research groups (range, 1–18 documents per group). The three most common types of documents were oral presentations (37%), journal articles (24%), and internal documents (22%). Four research groups (CHF, CMIMG, CPHG, EPOC) contributing 28 documents (68%) had primary affiliations associated with The Cochrane Collaboration. Eleven documents *described an author team*’*s experience conducting one or more overviews of healthcare interventions* (Additional file 3, references A42–A52). These documents (5 posters, 4 journal articles, 2 oral presentations) were produced between 2004 and 2015 by nine research groups (range, 1–2 documents per group). Six research groups (CCRG, CHF, CM, CS, DDC, EPOC) contributing 8 documents (73%) had primary affiliations associated with The Cochrane Collaboration. We first summarize the existing guidance for conducting overviews, with frequency and intensity effect sizes. We then summarize the challenges identified.

### Guidance for conducting overviews

The guidance contained within the 41 documents that *provided explicit methodological guidance* fell into two broad categories: guidance related to the context for conducting overviews and guidance related to the process of conducting overviews. These categories could be further subdivided into 15 topic areas. The existing methods guidance for each topic area is summarized below; italicized terms are defined in Table 3.

**Table 3:** Definitions

Indirect comparison: “A comparison of two interventions via one or more common comparators. For example, the combination of intervention effects from AC and intervention effects from BC studies may (in some situations) be used to learn about the intervention effect AB.” (http://methods.cochrane.org/cmi/node/61)	
Network meta-analysis: “An analysis that syntheses information over a network of comparisons to assess the comparative effects of more than two alternative interventions for the same condition. A network meta-analysis synthesizes direct and indirect evidence over the entire network, so that estimates of intervention effect are based on all available evidence for that comparisons. This evidence may be direct evidence, indirect evidence or mixed evidence. Typical outputs of a network meta-analysis are a) relative intervention effects for all comparisons; and b) a ranking of the interventions.” (http://methods.cochrane.org/cmi/node/61)	
Non-Cochrane systematic reviews: Systematic reviews published outside of the Cochrane Database of Systematic Reviews.	
Overlapping systematic reviews: Two or more systematic reviews examining the same intervention for the same disorder. Overlapping systematic reviews will often contain one or more of the same primary studies, which may lead to including the same study’s outcome data in an overview two or more times.	
Quality of evidence: The confidence we have in the outcome effect estimates, often assessed using the Grading of Recommendations Assessment, Development and Evaluation (GRADE) tool.	
Transitivity assumption: “The situation in which an intervention effect measured using an indirect comparison is valid and equivalent to the intervention effect measured using a direct comparison. Specifically, the transitivity assumption states that (the benefit of A over B) is equal to (the benefit of A over C) plus (the benefit of C over B). Equivalently, this may be written as (the benefit of A over C) minus (the benefit of B over C). In practice, transitivity requires similarity; that is that the sets of studies used to obtain the indirect comparison are sufficiently similar in characteristics that moderate the intervention effect. Transitivity can be thought of as a network meta-analysis extension of the idea of homogeneity in a standard meta-analysis.” (http://methods.cochrane.org/cmi/node/61)	

#### Guidance related to the context for conducting overviews

##### Choosing between conducting an overview and a SR

Two groups provided guidance on this topic (CMIMG, EPPI). The CMIMG stated that authors should conduct an overview only when they intend to search for and extract data from SRs as opposed to primary studies. Authors should conduct a SR when they intend to search for or extract data from primary studies, conduct a *network meta-analysis*, or rank order interventions (A16). When choosing between both study designs, the scope of the research question should be taken into account (A12). See reference A23 for additional guidance on choosing between both study designs (CMIMG). EPPI-Centre stated that authors may consider conducting an overview when a broad research question co-occurs with a short time frame and limited resources (A30).

##### What types of questions about healthcare interventions can be answered using the overview format?

One group (CMIMG) provided guidance on this topic, though six additional groups (CHF: A2; DukeU: A29; JBI: A34; NOKC: A36; TCD: A37; UBirm: A38) referenced this guidance in their own documents. The CMIMG stated that overviews can summarize evidence from multiple SRs about “different interventions for the same condition; the same intervention for different conditions; the same intervention for the same condition where different outcomes are addressed in different SRs; or adverse effects of interventions” (A16).

##### Questions to consider before deciding to conduct an overview

Six groups (CHF, CMIMG, EPPI, JBI, TCD, WJNR) stated that the overview format must be suitable for the proposed research question. Questions to consider include the following: is the topic clinically relevant (CHF: A8); is the field too new or changing too rapidly to preclude the utility of an overview (EPPI: A30); are there enough relevant SRs on major interventions and/or disorders of interest (e.g., are SRs up-to-date and clinically and methodologically homogeneous) (CHF: A8; CMIMG: A16; WJNR: A39); have important organizational factors been considered (e.g., author team, time frame, and funding) (CHF: A8; CMIMG: A14; JBI: A34; TCD: A37); and does it make methodological sense to include all SRs in the same overview (e.g., has the *transitivity assumption* been met) (CHF: A3; CMIMG: A24)? The CHF states that proper planning is important and that authors should “beware of the common misperception that overviews are easy and straightforward” (A3).

##### Author team composition and roles

Four groups (CHF, CMIMG, JBI, WJNR) stated that a complete multidisciplinary author team is needed that ideally includes a project coordinator (CHF: A4), clinician or content expert (CHF: A9), researcher with methodological expertise (CHF: A9; CMIMG: A20; JBI: A34; WJNR: A39), statistician (as needed) (CHF: A9; CMIMG: A20), and information specialist (as needed) (CHF: A9). Additional members may also be required, and roles should correspond to each member’s area of expertise (CHF: A9). See reference A9 for additional detail on team member roles (CHF).

##### Target audience of the overview

Eight groups (CHF, CMIMG, DukeU, EPOC, EPPI, TCD, WHU, WJNR) stated that the target audience for overviews is healthcare decision-makers including clinicians (CHF: A10; CMIMG: A16; EPOC: A27; TCD: A37; WHU: A41; WJNR: A39), researchers (DukeU: A29; EPOC: A27; WJNR: A39), informed patients/consumers (CHF: A10; CMIMG: A16; WHU: A41), and policy-makers/commissioning agents (CHF: A10; CMIMG: A16; EPOC: A27; EPPI: A30; WHU: A41; WJNR: A39).

#### Guidance related to the process for conducting overviews

##### Specifying the scope

Ten groups provided guidance on this topic (CHF, CMIMG, CPHG, DukeU, EPOC, EPPI, JBI, NOKC, TCD, WJNR). They stated that authors should clearly specify and describe the clinical characteristics (e.g., populations, interventions, comparators, and outcomes) and study design information (e.g., SRs) of interest for the overview (CHF: A8; CMIMG: A16; CPHG: A28; EPOC: A27; JBI: A34; NOKC: A36; TCD: A37; WJNR: A39). Reference A9 contains additional detail about specifying outcomes of interest (CHF). Additionally, authors may wish to restrict their scope based on clinical or methodological characteristics (CHF: A6; CMIMG: A16; DukeU: A29; EPOC: A27; EPPI: A30; JBI: A33; NOKC: A36; TCD: A37).

##### Searching for SRs

Eleven groups provided guidance on this topic (CHF, CMIMG, CPHG, DukeU, EPOC, EPPI, JBI, NOKC, TCD, WHU, WJNR). They stated that authors should search the CDSR to locate Cochrane SRs (CHF: A8; CMIMG: A16; EPPI: A30; JBI: A34; TCD: A37). To locate *non-Cochrane SRs*, authors should search additional databases (e.g., MEDLINE, EMBASE) and SR registries (e.g., Epistemonikos) (CHF: A8; CMIMG: A26; CPHG: A28; DukeU: A29; EPOC: A27; EPPI: A30; JBI: A34; TCD: A37; WJNR: A39) and contact experts or conduct handsearching of sources relevant to the topic (JBI: A34; TCD: A37). Overview authors may choose to use SR-specific search terms and/or validated SR search filters (CHF: A8; DukeU: A29; EPOC: A27; JBI: A34; TCD: A37). They may also restrict their search by date, language, and/or publication status, if appropriate (CPHG: A28; DukeU: A29; EPOC: A27; JBI: A34; TCD: A37). Conflicting guidance was provided regarding whether or not overview authors should search for and include primary studies that are not contained within any included SR (CHF: A2; CMIMG: A16; CPHG: A28; DukeU: A29; EPPI: A30; NOKC: A36; WHU: A41). Different ways of searching for primary studies were described, for example, see reference A41 (WHU).

##### Selecting SRs for inclusion

Six groups (CHF, CMIMG, DukeU, EPOC, NOKC, TCD) indicated that authors should select SRs for inclusion using pre-defined inclusion criteria. The scopes of the SRs and overview may sometimes differ (DukeU: A29; NOKC: A36); in these cases, authors must assess the primary studies contained within each SR for inclusion, and they should only include the subset of primary studies that meet the overview’s inclusion criteria (CHF: A8; CMIMG: A16). Two groups (EPOC: A27; TCD: A37) recommended that documents be assessed for inclusion by two independent reviewers with consensus.

##### Should an overview include non-Cochrane SRs?

Nine groups provided guidance on this topic (CHF, CMIMG, DukeU, EPOC, EPPI, JBI, NOKC, TCD, WHU). Two groups affiliated with The Cochrane Collaboration (CHF: A8; CMIMG: A16) stated that authors of Cochrane overviews should include only Cochrane SRs, if possible, but they also stated that including *non-Cochrane SRs* has both advantages (e.g., greater topic coverage) and disadvantages (e.g., increases complexity of the overview). The groups provided conflicting guidance regarding whether or not overview authors should use SR quality as an inclusion criterion for *non-Cochrane SRs* (and if so, what procedure to follow and which tool to use) (CHF: A8; DukeU: A29; EPOC: A27; EPPI: A30; JBI: A34; NOKC: A36; TCD: A37; WHU: A40). There was uncertainty and conflicting guidance on the methods that should be used to manage *overlapping SRs* in overviews (e.g., should authors include only one SR per topic area, or should they include all relevant SRs regardless of overlap?) (CHF: A8; CMIMG: A26; DukeU: A29; EPPI: A30; TCD: A37; WHU: A41). See reference A40 (WHU) for ways to assess and report overlap in overviews, and references A8 (CHF) and A29 (DukeU) for ways to potentially manage overlap in overviews.

##### Assessing quality of included SRs

All 12 groups stated that quality assessment of SRs is important and should be done. Conflicting guidance was provided regarding the tool that should be used, though A MeaSurement Tool to Assess systematic Reviews (AMSTAR) [20] was mentioned most often, by seven research groups (CHF: A8; CMIMG: A16; DukeU: A29; EPOC: A27; JBI: A33; TCD: A37; WJNR: A39). Six groups recommended dual independent quality assessments with consensus (CMIMG: A16; DukeU: A29; EPOC: A27; JBI: A34; NOKC: A36; TCD: A37). No other guidance was provided describing the specific methods that should be used to assess SR quality (e.g., whether and how to modify the quality assessment tool for use in overviews).

##### Collecting and presenting data on descriptive characteristics of included SRs (and their primary studies)

Six groups (CHF, CMIMG, EPOC, JBI, TCD, WJNR) provided guidance on this topic. They stated that authors should extract information about the objectives, inclusion criteria, and methods of each included SR (CHF: A8; CMIMG: A16; EPOC: A27; JBI: A33; TCD: A37; WJNR: A39). Authors should also extract information about the primary studies included in each SR (CHF: A8; EPOC: A27; JBI: A33; TCD: A37; WJNR: A39).

##### Collecting and presenting data on quality of primary studies contained within included SRs

Seven groups provided conflicting guidance regarding how overview authors should collect and present data on primary study quality; methods proposed included extracting and reporting the quality assessments conducted within each SR or referring back to each primary study to conduct quality assessments (CHF: A8; CMIMG: A16; DukeU: A29; EPPI: A30; JBI: A34; NOKC: A36; WJNR: A39). Four groups explicitly recommended the former method over the latter, if possible (CHF: A8; CMIMG: A16; JBI: A34; NOKC: A36). No guidance was provided regarding the logistical concerns likely to be encountered (e.g., use of different quality assessment tools in different SRs).

##### Collecting, analyzing, and presenting outcome data

Seven groups provided guidance on this topic and described quantitative and narrative methods of presenting data (CHF, CMIMG, DukeU, EPOC, EPPI, JBI, UBirm). Three groups (CHF: A8; DukeU: A29; UBirm: A38) stated that outcome data could be extracted from SRs and analyzed or presented in a different way than the analyses contained within the SRs (e.g., using meta-analysis or other complex methods). Two groups (CMIMG: A16; JBI: A33) stated that outcome data could simply be presented in the overview as they were presented in SRs. Two groups (EPOC: A27; EPPI: A30) acknowledged both approaches without recommending one over the other. Research groups advised that the most appropriate method of data analysis may depend upon the overview’s research question and the amount of clinical, methodological, and/or statistical heterogeneity in the SRs (CHF: A9; CMIMG: A12; EPPI: A30). Three groups recommended dual independent data extraction with consensus (EPOC: A27; JBI: A33; UBirm: A38). Research groups provided limited guidance regarding the logistical concerns likely to be encountered when analyzing outcome data. For example, there is uncertainty regarding how to analyze data from *overlapping SRs* (though at a minimum, authors should acknowledge the overlap and potential for bias) (CHF: A9; CMIMG: A26; EPPI: A30; JBI: A33; also see WHU: A40).

##### Assessing quality of evidence of outcome data

Six groups stated that it is important to assess the *quality of evidence*, for example, using the Grading of Recommendations Assessment, Development and Evaluation (GRADE) tool (CHF: A8; CMIMG: A16; EPPI: A30; JBI: A34; NOKC: A36; TCD: A37) [21]. However, only two groups provided guidance regarding how to assess quality of outcome data in overviews; they stated that authors could either extract quality assessments from included SRs or conduct quality assessments themselves at the overview level (CHF: A8; CMIMG: A16). CMIMG recommended that two reviewers independently assess *quality of evidence* with a process for consensus (A16). No other guidance was provided regarding the logistical concerns likely to be encountered when conducting quality assessments (e.g., what if not all SRs assessed *quality of evidence*?).

##### Interpreting outcome data and drawing conclusions

Three groups (CMIMG, EPPI, WHU) provided guidance on this topic. They stated that authors must ensure that the conclusions they make are warranted based on the quality of the primary studies and SRs and the methods used to analyze data (CMIMG: A16; EPPI: A30). Authors should avoid making informal *indirect comparisons* across different interventions because the *transitivity assumption* will likely be problematic (CMIMG: A24). Authors should also state whether more research is likely to change the results of the overview (based on *quality of evidence*, if assessed) (WHU: A41).

#### Frequency and intensity effect sizes

The research groups that contributed the most guidance to this scoping review, as measured using intensity effect sizes (Table 4), were as follows: CMIMG (15/15 topics), CHF (13/15 topics), and EPPI and JBI (11/15 topics each). The topic areas that the most research groups discussed, as measured using frequency effect sizes, were as follows: “assessing quality of SRs” (12/12 groups), “searching for SRs” (11/12 groups), and “specifying the scope” (10/12 groups). Topics that the least number of research groups discussed were as follows: “choosing between conducting an overview and a SR” (2/12 groups), “interpreting outcome data and drawing conclusions” (3/12 groups), and “author team composition and roles” (4/12 groups each).

**Table 4:** Map of methodological guidance for conducting overviews

Topic area	CHF	CMIMG	CPHG	DukeU	EPOC	EPPI	JBI	NOKC	TCD	UBirm	WHU	WJNR	Frequency effect size
Guidance related to the context for conducting overviews (i.e., when and why should you conduct an overview?)													
Choosing between conducting an overview and a SR		✓				✓							2/12
What types of questions about healthcare interventions can be answered using the overview format?	✓	✓		✓			✓	✓	✓	✓			7/12
Questions to consider before deciding to conduct an overview	✓	✓				✓	✓		✓			✓	6/12
Author team composition and roles	✓	✓					✓					✓	4/12
Target audience of the overview	✓	✓		✓	✓	✓			✓		✓	✓	8/12
Guidance related to the process of conducting overviews (i.e., how do you conduct an overview?)													
Specifying the scope	✓	✓	✓	✓	✓	✓	✓	✓	✓			✓	10/12
Searching for SRs	✓	✓	✓	✓	✓	✓	✓	✓	✓		✓	✓	11/12
Selecting SRs for inclusion	✓	✓		✓	✓			✓	✓				6/12
Should an overview include non-Cochrane SRs?	✓	✓		✓	✓	✓	✓	✓	✓		✓		9/12
Assessing quality of included SRs	✓	✓	✓	✓	✓	✓	✓	✓	✓	✓	✓	✓	12/12
Collecting and presenting data on descriptive characteristics of included SRs (and their primary studies)	✓	✓			✓		✓		✓			✓	6/12
Collecting and presenting data on quality of primary studies contained within included SRs	✓	✓		✓		✓	✓	✓				✓	7/12
Collecting, analyzing, and presenting outcome data	✓	✓		✓	✓	✓	✓			✓			7/12
Assessing quality of evidence of outcome data	✓	✓				✓	✓	✓	✓				6/12
Interpreting outcome data and drawing conclusions		✓				✓					✓		3/12
Intensity effect size	13/15	15/15	3/15	9/15	8/15	11/15	11/15	8/15	10/15	3/15	5/15	8/15	

### Challenges identified when conducting overviews

All 19 research groups contributing explicit guidance and/or author experiences identified at least one challenge involved when conducting overviews of healthcare interventions. These challenges are summarized in Table 5. Nine research groups also described limitations inherent to the overview format itself (CHF, CMIMG, CPHG, DukeU, EPOC, EPPI, JBI, WHU, WJNR). Specifically, they stated that overviews can be complex and resource intensive (CHF: A43; EPOC: A46; EPPI: A30; WHU: A40); susceptible to bias (CMIMG: A17; CPHG: A28; DukeU: A29; EPPI: A30; JBI: A34; WHU: A40); and dependent on (and limited by) the scope, inclusion criteria, methods, reporting, and coverage of their included SRs (CHF: A9; CPHG: A28; DukeU: A29; EPOC: A45; EPPI: A30; JBI: A34; WHU: A41; WJNR: A39). Few of the challenges identified when conducting overviews were adequately addressed by the methodological guidance previously summarized.

**Table 5:** Common challenges involved in conducting overviews

Topic area	Number of groups contributing challenges (/19)	Summary of challenges identified
Challenges related to the context for conducting overviews (i.e., when and why should you conduct an overview?)		
Choosing between conducting an overview and a SR	1 (CMIMG)	*Network meta-analyses* are very difficult to conduct in overviews and should likely not be conducted within overviews. It may be difficult to determine whether it is more appropriate to conduct an overview, or a systematic review with or without *network meta-analysis*.
What types of questions about healthcare interventions can be answered using the overview format?	2 (CCRG, CM)	Methods used to conduct overviews may vary according to the type of question (e.g., scope, clinical characteristics) being posed in the overview.
Questions to consider before deciding to conduct an overview	5 (CHF, CMIMG, DCC, JBI, UDun)	Should authors conduct an overview if there are not enough relevant SRs (e.g., if SRs do not address all important interventions)?
Author team composition and roles	2 (CHF, CMIMG)	Overview authors often have limited time. What skills are required for authors wishing to conduct overviews?
Target audience of the overview	0	No challenges identified.
Challenges related to the process of conducting overviews (i.e., how do you conduct an overview?)		
Specifying the scope	4 (EPPI, LBI, UBirm, UDun)	Defining the scope, and selecting and prioritizing outcomes, can be difficult. The scope of the overview may have almost complete overlap, or very limited overlap, with the scope of the relevant SRs.
Searching for SRs	5 (CHF, CPHG, EPOC, LBI, UBirm)	Search strategies can be complex. It is unclear whether government reports that include both primary studies and SRs should be included in an overview. It is unclear whether and how overview authors should search for primary studies that are not contained within any included SR.
Selecting SRs for inclusion	8 (CHF, CMIMG, DukeU, EPPI, JBI, UBirm, UDun, WHU)	It is unclear whether lower-quality SRs or older SRs should be included or excluded. Decisions surrounding inclusion and exclusion can affect the efficiency, utility, and breadth of the overview.
Should an overview include non-Cochrane SRs?	9 (CHF, CMIMG, CPHG, DukeU, EPOC, EPPI, TCD, WHU, WJNR)	Including *non-Cochrane SRs* can be difficult and will increase the complexity of the overview process. *Non-Cochrane SRs* can be of low methodological quality and may be poorly reported. Additionally, some Cochrane and *non-Cochrane SRs* will have overlap in their clinical questions, inclusion criteria, and/or included primary studies, and may have discordant results and/or conclusions. *Overlapping SRs* can be problematic, and there are potential challenges involved in assessing the amount of overlap in included SRs. Additionally, methods for choosing between *overlapping SRs* have not yet been developed; for example, it is unclear whether authors should include only one SR per topic area (and if so, which one?), or if they should include all SRs regardless of overlap (and if so, how will overlap be managed?). Authors including *non-Cochrane SRs* also have to clearly define what counts as a SR.
Assessing quality of included SRs	9 (CCRG, CHF, CMIMG, CPHG, EPOC, EPPI, PXU, UBirm, UDun)	Assessing quality of SRs can be difficult and time-consuming. Many different tools could be used to assess SR quality, and some tools designed to assess quality may also assess reporting. There is also uncertainty surrounding how to interpret and apply the results of quality assessments in the context of overviews.
Collecting and presenting data on descriptive characteristics of included SRs (and their primary studies)	11 (CCRG, CHF, CM, CMIMG, DCC, DukeU, EPOC, JBI, LBI, UDun, NOKC)	Data may be missing, inadequately reported, or reported differently across included SRs, and it is unclear what to do when reporting is incomplete (e.g., should the data be extracted from primary studies?). Additionally, data extraction errors in SRs could lead to errors in the overview.
Collecting and presenting data on quality of primary studies contained within included SRs	7 (CCRG, CHF, CM, DCC, EPOC, EPPI, UDun)	Collecting and presenting quality of primary studies can be difficult and time-intensive. Information about the quality of primary studies included in SRs may be missing, inadequately reported, or reported differently across included SRs. For example, different SRs may use different tools to assess quality of primary studies.
Collecting, analyzing, and presenting outcome data	15 (CCRG, CHF, CM, CMIMG, DCC, DukeU, EPOC, EPPI, JBI, LBI, NOKC, UBirm, UDun, WJNR, WHU)	Collecting, analyzing and presenting outcome data can be difficult, especially when the scope, methods, or results of the included SRs are heterogeneous. Outcome data may be missing, inadequately reported, or reported inconsistently across included SRs, and it is unclear what to do in these situations (e.g., should the data be extracted from primary studies instead?). It is also unclear how best to summarize and report outcome data that comes from *overlapping* (and potentially discordant) *SRs*. It may not always be possible or appropriate to conduct meta-analyses in overviews or to directly compare results across different SRs. Similarly, *network meta-analyses* are often not appropriate in overviews. Additionally, overviews may not accurately capture information about adverse effects or cost-effectiveness of interventions, and data extraction errors in SRs could lead to errors in the overview.
Assessing quality of evidence of outcome data	9 (CCRG, CHF, CM, CPHG, DCC, EPOC, PXU, UDun, WHU)	It may not be possible to simply extract existing GRADE assessments from SRs. However, it may be challenging to conduct (or re-do) GRADE assessments at the overview level, using data from SRs. For example: data needed to assess *quality of evidence* in SRs may be missing, inadequately reported, and/or reported differently across included SRs; the “study quality” domain may be assessed differently across similar SRs (e.g., different tools used, same tool used but different assessments obtained, only summary assessments reported); and the “consistency” and “precision” domains may be affected if different methodological decisions are made in similar SRs (e.g., pooling versus not pooling data). Additionally, achieving consensus may be difficult. The GRADE tool may need to be modified for use in overviews.
Interpreting outcome data and drawing conclusions	6 (CHF, CMIMG, DukeU, EPOC, LBI, WJNR)	Interpreting outcome data and drawing conclusions can be difficult. There is uncertainty surrounding how to interpret outcome data in overviews. It can be difficult to form a coherent judgment when multiple different comparisons from multiple SRs are included in the same overview, and/or when *overlapping SRs* report discordant results. It can also be difficult to determine implications for research. Additionally, there is concern that the methods used to conduct overviews might affect the conclusions reached.

*CDSR* Cochrane Database of Systematic Reviews, *DARE* Database of Abstracts of Reviews of Effectiveness, *EMBASE* Excerpta Medica dataBASE, *GRADE* Grading of Recommendations Assessment, Development and Evaluation, *PICO* populations, interventions, comparators, and outcomes, *SR* systematic review

## Discussion

This scoping review found relatively consistent and comprehensive guidance for the first stages of the overview process, from choosing to conduct an overview through to selecting SRs for inclusion. Guidance for the latter stages was often conflicting and/or missing, and a number of outstanding challenges were identified. These latter stages included the following: deciding whether to include SRs published outside of the CDSR, assessing quality of SRs and their primary studies, collecting and analyzing data, and assessing quality of evidence of outcome data.

The shift from consistent to conflicting and/or missing guidance that occurs after the inclusion stage may be due to several factors. First, this is the point at which guidance for overviews takes on an additional level of complexity. Within an overview, there are two levels for assessing and reporting SR/study characteristics, quality/risk of bias, and outcome data (i.e., for both the SRs and their included primary studies). Existing methodological guidance does not yet adequately address how these stages of the overview process should occur relative to these two levels of information. Second, SRs are syntheses of pre-existing data, and we found that overviews are limited by the methods and reporting of their included SRs. Data may be missing, inadequately reported, or reported differently across included SRs, and it is currently unclear whether overview authors should rely solely on the SRs as they were conducted and presented, or whether and to what extent authors should refer back to the primary studies for additional information. Lastly, including SRs published outside of the CDSR can increase the complexity of the latter stages of the overview process due to greater variation in the methods and reporting of non-Cochrane SRs [22, 23] and the potential for topic overlap across multiple similar SRs [24]. Limited guidance was available regarding the specific methods authors can use to address and manage these issues in overviews.

To circumvent some of the challenges authors are likely to encounter during the latter stages of the overview process, authors should first ensure that the overview format is appropriate for their question of interest. The CMIMG in particular provided comprehensive guidance regarding the context for conducting an overview (i.e., when and why to conduct an overview); however, much of this guidance is currently in the form of internal documents and conference proceedings that may be difficult for authors to access. Authors should also prepare a detailed protocol for their overview. Often overview authors describe their scope and inclusion criteria but provide less detail about methods to be used for quality assessments and data extraction and analysis. As well as reducing bias and promoting rigor and transparency of methods [11, 25], a protocol would allow overview authors to become familiar with the challenges they are likely to encounter and develop a priori decision rules to appropriately address those challenges. The guidance and challenges described in this paper will be useful for authors to consider when developing their protocols.

As is common when using a qualitative metasummary approach [18], an important insight emerged when we analyzed our data across topic areas, namely, that overviews are often conducted for one of two main purposes. The first purpose is to present and describe the complete body of SR evidence on a clearly defined topic [26, 27]. The second purpose is to address a question that differs from the question(s) in the underlying SRs and that often relates to a subset of the questions in the SRs (e.g., subpopulations, or subsets of interventions or outcomes) [28, 29]. Distinguishing between these two purposes, and recognizing that different methods may be used for each, can help resolve some discrepancies and challenges likely to be encountered during the latter stages of the overview process. For example, if the purpose is to answer a different question from those posed in the SRs, authors may wish to re-extract and re-analyze outcome data (e.g., using meta-analysis) from a set of non-overlapping SRs. However, if the purpose is to describe the complete body of SR evidence on a topic, authors may find it more appropriate to include all relevant SRs regardless of topic overlap and then present these results as they appeared in the SRs. Empirical evidence will be needed to determine whether these approaches affect the results or introduce bias at the overview level.

Ultimately, methodological guidance is required for those stages of the overview process where guidance is conflicting and/or missing and where outstanding challenges remain. This future guidance should be based on empirical evidence generated from well-conducted studies that evaluate methods for conducting overviews. While outside of the scope of the present paper, we identified several relevant methods studies (recently published, and in progress) when conducting this scoping review. These methods studies examined the following: implications of including multiple SRs published on the same topic area [24]; issues related to quality assessment of SRs [30–32]; different methods for presenting outcome data [33]; methods for assessing quality of evidence using GRADE [34, 35]; and reporting conflicts of interest in overviews [36]. One additional study (in progress) was identified that will aim to summarize all empirical studies [37]. Developing future methodological guidance for overviews based on the results of empirical studies will help ensure that guidance is based on sound evidence as opposed to personal experience or trial and error.

The current scoping review aimed to identify and collate all documents containing methodological guidance for conducting overviews of healthcare interventions. Due to the variety of publication formats for overview methods guidance and the difficulty in locating and accessing these documents, it is possible we may have missed relevant guidance documents. To maximize retrieval, our search utilized multiple complimentary methods in addition to database searching. We had satisfactory response rates (ranging from 55 to 96%) when locating and obtaining the full text of unpublished documents, and we were able to translate and extract data from all relevant non-English documents identified. We then used a rigorous process for identifying, extracting, and analyzing guidance statements and challenges from these documents. Importantly, we were interested in method guidance and challenges for overviews examining the efficacy, effectiveness, and/or safety of healthcare interventions and have excluded guidance and challenges specific to overviews that may address broader or different clinical questions. Guidance for conducting these other types of overviews is also needed but is outside the scope of the current project. It is important to note that the guidance and challenges summarized here was written by research groups with different organizational processes that likely produce overviews with differing purposes, scopes, target audiences, and/or resource requirements. Researchers should identify the purpose, scope, target audience, and resource requirements of their overview at the outset and determine how well the guidance and challenges presented here apply to their specific situation. Lastly, the guidance included in this scoping review came from documents that explicitly *intended* to provide methods guidance to readers: the methods presented here do not come from the actual methods used in published overviews. However, discussions with overview authors [13] and critical appraisal of published overviews [4, 5, 38, 39] indicates that the guidance and challenges in this scoping review are congruent with overview authors’ experiences.

## Conclusions

This scoping review provides a systematic summary of existing methodological guidance for conducting overviews examining the efficacy, effectiveness, and/or safety of healthcare interventions. It highlights the stages of the overview process where guidance is consistent, conflicting, or missing, and it also provides a summary of the challenges involved in conducting overviews. This scoping review will serve as a useful resource for authors wishing to conduct overviews, as well as researchers wishing to conduct empirical research on overview methods. It is also a necessary first step to developing a cohesive methods guidance document that addresses relevant issues and areas of uncertainty when conducting overviews of healthcare interventions. Accordingly, the results of this scoping review were used to update the chapter on overview methods in the *Cochrane Handbook*. There has been a dramatic rise in the production of SRs and overviews in recent years. These syntheses are an important vehicle to increase the uptake and application of knowledge by clinical and policy decision-makers, and they can help address crucial health issues and ultimately improve health outcomes in diverse populations. Investing in strengthening the methods guidance for conducting overviews can help ensure a rigorous and valid evidence base for knowledge translation and dissemination.

## Additional files

Additional file 1: Complete search strategies. (DOCX 36.0 kb)
Additional file 2: Text extracted and not extracted from included documents. (DOCX 16.0 kb)
Additional file 3: Included documents. (DOCX 28.0 kb)

## Abbreviations

AMSTAR: A MeaSurement Tool to Assess systematic Reviews
CCRG: Cochrane Consumers and Communication Review Group
CDSR: Cochrane Database of Systematic Reviews
CHF: Cochrane Child Health Field
CM: Cochrane Musculoskeletal Review Group
CMIMG: Cochrane Comparing Multiple Interventions Methods Group
CPHG: Cochrane Public Health Group
CS: Cochrane Stroke Group
DARE: Database of Abstracts of Reviews of Effects
DCC: Dutch Cochrane Centre
DukeU: Duke University
EMBASE: Excerpta Medica dataBASE
EPOC: Cochrane Effective Practice and Organization of Care Review Group
EPPI: Evidence for Policy and Practice Information and Co-ordinating Centre
GRADE: Grading of Recommendations Assessment, Development and Evaluation
JBI: Joanna Briggs Institute Umbrella Reviews Methodology Group
LBI: Ludwig Boltzmann Institute for Health Technology Assessment
NOKC: Norwegian Knowledge Centre for the Health Services
Overview: Overview of reviews
PXU: Pontifical Xavierian University
SR: Systematic Review
TCD: Trinity College Dublin
UBirm: University of Birmingham
UDun: University of Dundee
WHU: Witten/Herdecke University
WJNR: Western Journal of Nursing Research
