# Supplementary material for: What guidance is available for researchers conducting overviews of reviews of healthcare interventions? A scoping review and qualitative metasummary
Source: Syst Rev. 2016 Nov 14;5:190. doi: 10.1186/s13643-016-0367-5 (PMC5109841; doi:10.1186/s13643-016-0367-5)
Supplement: Additional file 1: — Complete search strategies. (DOCX 36.0 kb) [file 13643_2016_367_MOESM1_ESM.docx]

**Additional file 1: Complete search strategies**

**1. Reference Tracking**

Start Date: 21 January 2014

End Date: 14 March 2014

Procedure*: For each target article, we searched for "citing" references (Google Scholar), "cited" references (Scopus, reference lists), and "similar articles" (PubMed).

Target Articles (30):

1. Agency for Quality and Accreditation in Health Care Croatia. The Croatian guideline for health technology assessment process and reporting (1st edition). Zagreb; 2011.
2. Becker L, Caldwell D, Higgins J, Li T, Salanti G, Schmid C. Comparing multiple interventions in Cochrane reviews. In: Comparing multiple interventions in Cochrane reviews. Cochrane Comparing Multiple Interventions Methods Group. 2013. http://www. cmim.cochrane.org/comparing-multiple-interventions-cochrane-reviews [Follow link saying "A background paper explaining the rationale"]. Last accessed 30 Nov 2015.
3. Becker LA, Oxman AD. Chapter 22: overviews of reviews. In: Higgins JPT, Green S, editors. Cochrane handbook for systematic reviews of interventions (version 5.1.0). The Cochrane Collaboration; 2011.
4. Caldwell DM, Welton NJ, Ades AE. Mixed treatment comparison analysis provides internally coherent treatment effect estimates based on overviews of reviews and can reveal inconsistency. J Clin Epidemiol. 2010;63(8):875-82.
5. Centre for Reviews and Dissemination. Systematic reviews: CRD's guidance for undertaking reviews in health care. York: University of York; 2009.
6. Cleemput I, Van Den Bruel A, Kohn L, Vlayen J, Vinck I, Thiry N, et al. Search for evidence & critical appraisal: health technology assessment (HTA) (version 2007-1.1). Brussels: Belgian Health Care Knowledge Centre; 2007.
7. Conn VS, Coon Sells TG. WJNR welcomes umbrella reviews. West J Nurs Res. 2014;36(2):147-51.
8. Cooper H, Koenka AC. The overview of reviews: unique challenges and opportunities when research syntheses are the principal elements of new integrative scholarship. Am Psychol. 2012;67(6):446-62.
9. Delgado-Rodriguez M. Systematic reviews of meta-analyses: applications and limitations. J Epidemiol Commun H. 2006;60:90-92.
10. Elliott L, Crombie IK, Irvine L, Cantrell J, Taylor J. The effectiveness of public health nursing: the problems and solutions in carrying out a review of systematic reviews. J Adv Nurs. 2004;45(2):117-25.
11. Froschl B, Brunner-Ziegler S, Conrads-Frank A, Eisenmann A, Gartlehner G, Grillich L, et al. Methodenhandbuch fur health technology assessment (vorab-version 1.2012) [Methods manual for health technology assessment (pre-version 1.2012)]. Vienna: Health Austria GmbH; 2012.
12. Hartling L, Vandermeer B, Fernandes RM. Systematic reviews, overviews of reviews and comparative effectiveness reviews: a discussion of approaches to knowledge synthesis. Evid Based Child Health. 2014;9(2):486-94.
13. Hartling L, Chisholm A, Thomson D, Dryden DM. A descriptive analysis of overviews of reviews published between 2000 and 2011. PLoS One. 2012;7(11):e49667.
14. Hartling L, Vandermeer B, Moher D, Caldwell D, Dryden D. Optimizing evidence synthesis for informed decision-making. Canadian Institutes of Health Research Operating Grant. 2011.
15. Healthcare Improvement Scotland. Standard operating procedure for production of evidence notes. 2012.
16. Hemming K, Bowater RJ, Lilford RJ. Pooling systematic reviews of systematic reviews: a Bayesian panoramic meta-analysis. Stat Med. 2012;31(3):201-16.
17. Ludwig Boltzmann Gesellschaft Institute for Health Technology Assessment. (Internes) Manual ablaufe und methoden (teil 2) [(Internal) Manual of processes and methods (part 2)]. Vienna: Ludwig Boltzmann Gesellschaft GmbH; 2007.
18. Institute for Quality and Efficiency in Health Care. Allgemeine methoden (version 4.0) [General methods (version 4.0)]. Cologne; 2011.
19. Ioannidis JPA. Integration of evidence from multiple meta-analyses: a primer on umbrella reviews, treatment networks and multiple treatments meta-analyses. Can Med Assoc J. 2009;181(8):488-93.
20. Li L, Tian J, Tian H, Sun R, Liu Y, Yang K. Quality and transparency of overviews of systematic reviews. J Evid Based Med. 2012;5(3):166-73.
21. National Institute for Health and Clinical Excellence. Methods for the development of NICE public health guidance (third edition). 2012.
22. Pertl D, Froschl B, Rosian-Schikuta I, Sturzlinger H, Freiberger I. Prozesshandbuch für health technology assessment (Bundesinstitut fur Qualita im Gesundheitswesen) [Process manual for health technology assessment (Federal Institute for Quality in Health Care)]. Vienna: Health Austria GmbH; 2010.
23. Pieper D, Antoine SL, Mathes T, Neugebauer EA, Eikermann M. Systematic review finds overlapping reviews were not mentioned in every other overview. J Clin Epidemiol. 2014;67(4):368-75.
24. Peiper D, Buchter RB, Antoine SL, Eikermann M. Overviews - status quo, potentiale und ausblick [Overviews - status quo, potentials and perspectives]. Z Evid Fortbild Qual Gesundhwes. 2013;107:592-6.
25. Pieper D, Antoine SL, Morfeld JC, Mathes T, Eikermann M. Methodological approaches in conducting overviews: current state in HTA agencies. Res Synth Methods. 2013;5(3):187-99.
26. Pieper D, Buechter R, Jerinic P, Eikermann M. Overviews of reviews often have limited rigor: a systematic review. J Clin Epidemiol. 2012;65(12):1267-73.
27. Ryan RE, Kaufman CA, Hill SJ. Building blocks for meta-synthesis: data integration tables for summarising, mapping, and synthesising evidence on interventions for communicating with health consumers. BMC Med Res Methodol. 2009;9:16.
28. Smith V, Devane D, Begley CM, Clarke M. Methodology in conducting a systematic review of systematic reviews of healthcare interventions. BMC Med Res Methodol. 2011;11(1):15.
29. Thomson D, Foisy M, Oleszczuk M, Wingert A, Chisholm A, Hartling L. Overview of reviews in child health: evidence synthesis and the knowledge base for a specific population. Evid Based Child Health. 2013;8(1):3-10.
30. Thomson D, Russell K, Becker LA, Klassen T, Hartling L. The evolution of a new publication type: steps and challenges of producing overviews of reviews. Res Synth Meth. 2010;1(3-4):198-211.

* Procedure modified from: Horsley T, Dingwall O, Sampson M. Checking reference lists to find additional studies for systematic reviews. Cochrane Database Syst Rev. 2011;(8):MR000026. Greenhalgh T, Peacock R. Effectiveness and efficiency of search methods in systematic reviews of complex evidence: audit of primary sources. BMJ. 2005;331(7524):1064-5.

**2. Database and Web Searches**

Database: Medline via Ovid (1946 to Present)

Search Date: 17 January 2014

Search Results:

| 1. ((overview adj3 reviews) or (overview adj2 review*)).tw.  2. (umbrella adj5 review*).tw.  3. (systematic adj3 overview*).tw.  4. (overview adj2 cochrane adj2 reviews).tw.  5. (systematic adj1 reviews).ti.  6. ((appraisal or analysis or results) adj2 systematic adj review*).tw.  7. (meta-synthesis or (meta adj synthesis)).tw.  8. (meta-review or (meta adj review)).tw.  9. or/1-8 (3561)  10. limit 9 to (english language and humans and yr="2010 - 2013") (994) |
| --- |

Database: EMBASE via Ovid (1996 to 2014 Week 03)

Search Date: 17 January 2014

Search Results:

| 1. ((overview adj3 reviews) or (overview adj2 review*)).tw.  2. (umbrella adj5 review*).tw.  3. (systematic adj3 overview*).tw.  4. (overview adj2 cochrane adj2 reviews).tw.  5. (systematic adj1 reviews).ti.  6. ((appraisal or analysis or results) adj2 systematic adj review*).tw.  7. (meta-synthesis or (meta adj synthesis)).tw.  8. (meta-review or (meta adj review)).tw.  9. or/1-8 (4335)  10. limit 9 to (english language and humans and yr="2010 - 2013") (1468) |
| --- |

Database: Database of Abstracts of Reviews of Effects (DARE) via Cochrane Library (Issue 4 of 4, October 2013)

Search Date: 17 January 2014

Search Results:

| 1. (overview near/3 reviews) or (overview near/2 review*)  2. (umbrella near/5 review*)  3. (systematic near/3 overview*)  4. (overview near/2 cochrane near/2 reviews)  5. ((appraisal or analysis or results) near/2 systematic near/1 review)  6. (meta-synthesis or (meta next synthesis))  7. (meta-review or (meta next review))  8. #1 or #2 or #3 or #4 or #5 or #6 or #7 (1604)  9. Limit #8 to “Other Reviews” (288)  10. Limit #9 to “2010-2013” (112) |
| --- |

Database: Scopus

Search Date: 17 January 2014

Search Results:

| TITLE((overview PRE/3 reviews) OR (umbrella PRE/2 review*) OR (systematic PRE/2 overviews) OR (overview PRE/2 cochrane PRE/1 reviews) OR meta-review) OR ABS((overview PRE/3 reviews) OR (umbrella PRE/2 review*) OR (systematic PRE/2 overviews) OR (overview PRE/2 cochrane PRE/1 reviews) OR meta-review) AND LANGUAGE(english) AND (LIMIT-TO(PUBYEAR, 2010) OR LIMIT-TO(PUBYEAR, 2011) OR LIMIT-TO(PUBYEAR, 2012) OR LIMIT-TO(PUBYEAR, 2013)) AND (LIMIT-TO(EXACTKEYWORD, "Human") OR LIMIT-TO(EXACTKEYWORD, "Humans")) (324) |
| --- |

Database: Medline via Web of Science*

Search Date: 21 January 2014 (search strategy, except for date restriction, was then turned into an article alert; alerts were monitored as part of the update search up to 09 November 2015)

Search Results:

| (1.) TI="analys* of systematic reviews"  (2.) TI="guideline* based on systematic review*"  (3.) TI="overview* of Cochrane and non-Cochrane reviews"  (4.) TI="overview* of Cochrane reviews"  (5.) TI="overview* of Cochrane systematic reviews"  (6.) TI="overview* of review*"  (7.) TI="overview* of systematic reviews"  (8.) TI="review* of meta-analyses"  (9.) TI="review* of reviews"  (10.) TI="review* of systematic reviews"  (11.) TI="summar* of Cochrane"  (12.) TI="summar* of systematic reviews"  (13.) TI="synops* of Cochrane systematic reviews"  (14.) TI=“systematic review* of meta-analyses”  (15.) TI=“systematic review* of reviews”  (16.) TI =“systematic review* of systematic reviews”  (17.) #16 OR #15 OR #14 OR #13 OR #12 OR #11 OR #10 OR #9 OR #8 OR #7 OR #6 OR #5 OR #4 OR #3 OR #2 OR #1 (266)  (18.) #17 Indexes=MEDLINE Timespan=2010-2013 (158) |
| --- |

### * Medline via Web of Science was searched in addition to Medline via Ovid because the interface allows stop words (e.g., of, on, etc.).

Database: Cochrane Methods Studies Database via Cochrane Library

Search Date: 17 January 2014

Search Results:

| 1. "overview* of cochrane reviews":ti,ab,kw  2. "overview* of reviews":ti,ab,kw  3. "overview* of systematic reviews":ti,ab,kw  4. "review* of meta-analyses":ti,ab,kw  5. "review* of reviews":ti,ab,kw  6. "review* of systematic reviews":ti,ab,kw  7. "summar* of systematic reviews":ti,ab,kw  8. "systematic review* of reviews":ti,ab,kw  9. "systematic review* of systematic reviews":ti,ab,kw  10. "analys* of systematic reviews":ti,ab,kw  11. “umbrella review*”:ti,ab,kw  12. “systematic overview*”:ti,ab,kw  13. meta-synthes*:ti,ab,kw  14. meta-review*:ti,ab,kw  15. #1 or #2 or #3 or #4 or #5 or #6 or #7 or #8 or #9 or #10 or #11 or #12 or #13 or #14 from 2010 to 2014 (189)  Limit #15 to Methods Studies (85) |
| --- |

Website: Google Scholar

Search Date: 17 March 2014 (search strategy was then turned into an article alert; alerts were monitored as part of the update search up to 09 November 2015)

Results:

| (”review of reviews”\|”overview of systematic reviews”\|”review of systematic reviews”\|”systematic review of reviews”\|”overview of reviews”\|”umbrella review”\|”systematic overview”) (200)* |
| --- |

* The first 20 pages of results were reviewed.

**3. Handsearching Websites and Conference Proceedings**

Search Date (Websites): 17 March 2014

Websites Searched (26):

| **Website** | **URL** |
| --- | --- |
| Alberta Heritage Foundation for Medical Research | <http://www.aihealthsolutions.ca/> |
| Canadian Agency for Drugs and Technologies in Health | <http://www.cadth.ca/> |
| Canadian Institutes of Health Research | <http://www.cihr-irsc.gc.ca/e/193.html> |
| Centro Cochrane do Brasil | <http://www.centrocochranedobrasil.org.br/cms/> |
| Cochrane Child Health Field | <http://childhealth.cochrane.org/> |
| Cochrane Comparing Multiple Interventions Methods Group | <http://cmim.cochrane.org/> |
| Cochrane Consumers and Communications Group | <http://cccrg.cochrane.org/> |
| Cochrane Effective Practice and Organization of Care | <http://epoc.cochrane.org/> |
| Cochrane Hepato-Biliary Group | <http://hbg.cochrane.org/> |
| Cochrane Incontinence Group | <http://incontinence.cochrane.org/> |
| Cochrane Musculoskeletal Group | <http://musculoskeletal.cochrane.org/> |
| Cochrane Schizophrenia Group | <http://szg.cochrane.org/> |
| Comprehensive Cancer Centre South, The Netherlands | <http://www.eindhovencancerregistry.nl/page.php?id=3527&nav_id=97> |
| Department of Violence and Injury Prevention and Disability, World Health Organization | <http://www.who.int/violence_injury_prevention/en/> |
| Finnish Office for Health Technology Assessment and National Research and Development Centre for Welfare and Health | <http://www.thl.fi/en_US/web/en> |
| Health Protection Scotland | <http://www.hps.scot.nhs.uk/> |
| Iberoamerican Cochrane Group | <http://es.cochrane.org/es> |
| Institute for Clinical Effectiveness and Health Policy, Argentina | <http://www.iecs.org.ar/index.php> |
| Joanna Briggs Institute | <http://joannabriggs.org/> |
| McMaster University Health Systems Evidence. | <http://www.healthsystemsevidence.org> |
| Netherlands Institute for Health Services Research | <http://www.nivel.nl/en> |
| Norwegian Knowledge Centre for the Health Services | <http://www.kunnskapssenteret.no/home> |
| South African Cochrane Centre | <http://www.mrc.ac.za/cochrane/> |
| Netherlands Institute of Mental Health and Addiction | <http://www.trimbos.org/> |
| UK Cochrane Centre | <http://ukcc.cochrane.org/about-uk-cochrane-centre> |
| Workers' Compensation Board Evidence Based Practice Group, Workers' Compensation Board of BC | <http://worksafebc.com/health_care_providers/related_information/evidence_based_medicine/default.asp> |

Search Date (Conference Proceedings): 03 March 2014 (Conference Years: 2000-2013)

Search Date for Update Search: 09 November 2015 (Conference Years: 2014-2015)

Conference Proceedings Searched (3):

| **Conference Name** | **URL** |
| --- | --- |
| International Cochrane Colloquium | <http://www.abstracts.cochrane.org> |
| Health Technology Assessment (HTA) International | <http://www.htai.org/meetings/annual-meetings/past-annual-meetings.html> |
| Canadian Agency for Drugs and Technologies in Health (CADTH) Symposium | <https://www.cadth.ca/cadth-symposium-archives>  (also used general web searches) |

Note: HTA International could only be searched between 2007-2015 and CADTH Symposium could only be searched between 2005-2015.

**4. Contacting Producers of Overviews**

Date Contacted: April 15, 2014

Date Contacted for Update Search: 09 November 2015

Results:

| **Type of overview producer** | **Number contacted (number contacted for update search)** |
| --- | --- |
| Managing Editors of Cochrane Review Groups and Fields | 20 (5) |
| Authors of published overviews* | 110 (0) |

* Lists of authors were obtained from: Hartling L, Chisholm A, Thomson D, Dryden DM. A descriptive analysis of overviews of reviews published between 2000 and 2011. PLoS One. 2012;7(11):e49667. Pieper D, Buechter R, Jerinic P, Eikermann M. Overviews of reviews often have limited rigor: a systematic review. J Clin Epidemiol. 2012;65(12):1267-73.
