# Supplementary material for: What guidance is available for researchers conducting overviews of reviews of healthcare interventions? A scoping review and qualitative metasummary
Source: Syst Rev. 2016 Nov 14;5:190. doi: 10.1186/s13643-016-0367-5 (PMC5109841; doi:10.1186/s13643-016-0367-5)
Supplement: Additional file 2: — Text extracted and not extracted from included documents. (DOCX 16.0 kb) [file 13643_2016_367_MOESM2_ESM.docx]

**Additional file 2: Text extracted and not extracted from included documents**

| **Type of guidance document** | **Text targeted for extraction** | **Text not eligible for extraction** |
| --- | --- | --- |
| *Provides explicit methodological guidance for conducting overviews of healthcare interventions.* | Any text that *provides guidance or advice* to help overview authors conduct any part of an overview of healthcare interventions. This includes guidance related to the *context* for conducting overviews (i.e., when and why should researchers conduct an overview?) and guidance related to the *process* of conducting overviews (i.e., how should researchers conduct an overview?). | 1) Guidance or advice on how to conduct *other types of overviews* (e.g., diagnostic test accuracy, prognostic, and qualitative overviews).  2) *Examples from published overviews* showing how the guidance can be put into practice.  3) Statements explaining the *rationale* behind, or the *importance* of, the guidance.  4) Descriptions of guidance that originate from *other guidance documents* already included in this scoping review.  5) Guidance statements relating to *organizational structures and/or processes* of overview-producing organizations (e.g., The Cochrane Collaboration).  6) Guidance or advice on how to *report, peer review, or critically appraise* any part of an overview. |
|  | Any text that *describes challenges involved* when conducting an overview of healthcare interventions, regardless of whether or not specific guidance or advice is provided on how to address the challenge. | 1) Descriptions of challenges specific to conducting other types of overviews (e.g., diagnostic test accuracy, prognostic, and qualitative overviews).  2) *Content from published overviews* showing examples of the challenge encountered.  3) Explanations describing *why* the challenge was encountered or *how* the challenge was resolved.  4) Descriptions of challenges that originate from *other documents* already included in this scoping review.  5) Text that describes challenges author teams encountered when *reporting, peer reviewing, or critically appraising* any part of an overview. |
| *Describes an author team's experience conducting one or more overviews of healthcare interventions.* | Any text that *describes challenges author teams encountered* when conducting an overview of healthcare interventions, regardless of whether or not specific guidance or advice is provided on how to address the challenge. | See above. |
