# Supplementary material for: What guidance is available for researchers conducting overviews of reviews of healthcare interventions? A scoping review and qualitative metasummary
Source: Syst Rev. 2016 Nov 14;5:190. doi: 10.1186/s13643-016-0367-5 (PMC5109841; doi:10.1186/s13643-016-0367-5)
Supplement: Additional file 3: — Included documents. (DOCX 28.0 kb) [file 13643_2016_367_MOESM3_ESM.docx]

**Additional file 3: Included documents**

**Documents that contain explicit methodological guidance for conducting overviews (41 documents produced by 12 research groups)**

1. Becker LA, Thomson D, Caldwell D. Addressing multiple treatments: i - Cochrane overviews. Abstracts of the Joint Colloquium of The Cochrane and Campbell Collaborations, 18-22 Oct 2010. Keystone, USA: John Wiley & Sons; 2010. [Available online: http://www.cmim. cochrane.org/keystone-2010. Accessed 30 Nov 2015.]
2. Foisy M. An introduction to overviews of reviews (umbrella reviews). Northern Alberta Health Libraries Association Leading Edge Symposium, 26 Nov 2014. Edmonton, Canada.
3. Foisy M, Thomson D, Dryden DM, Fernandes RM, Hartling L. Conducting overviews of reviews: lessons learned since 2006. Abstracts of the 22nd Cochrane Colloquium, 21-26 Sep 2014. Hyderabad, India: John Wiley & Sons; 2014.
4. Foisy M, Thomson D, Dryden DM, Hartling L. Overviews of reviews: a new publication type and an emerging method of knowledge synthesis. Connecting Through Research Pediatric Research Day, 30 May 2014. Edmonton, Canada.
5. Foisy M, Thomson D, Dryden DM, Hartling L. Overviews of reviews: a new publication type and an emerging method of knowledge synthesis. Women and Children's Health Research Institute Research Day, 12 Nov 2014. Edmonton, Canada.
6. Hartling L, Fernandes R, Becker L, Foisy M. Comparing multiple treatments: an introduction to overviews of reviews. Abstracts of the 23rd Cochrane Colloquium, 03-07 Oct 2015. Vienna, Austria: John Wiley & Sons; 2015.
7. Thomson D, Becker LA, Foisy M. A primer to Cochrane overviews of reviews. Cochrane Canada Live Webinars, 06 Jun 2011. Online. [Available online: http://www.youtube.com/ watch?v=HzSPAvpWpl8. Accessed 30 Nov 2015.]
8. Thomson D, Foisy M, Hartling L. Overviews of reviews: what they are, what they aren't and how and when to do one. Cochrane Canada Live Webinars, 05 Dec 2013. Online. [Available online: http://www.youtube.com/watch?v=kj4aA2wPIRs. Accessed 30 Nov 2015.]
9. Thomson D, Russell K, Becker LA, Klassen T, Hartling L. The evolution of a new publication type: steps and challenges of producing overviews of reviews. Res Synth Meth. 2010;1(3-4):198-211.
10. Cochrane Child Health. Getting started on an overview of reviews. n.d. [Unpublished.]
11. Cochrane Child Health. Notes on the process of preparing an umbrella review for Evidence-Based Child Health. n.d. [Unpublished.]
12. Becker L, Caldwell D. Comparing multiple treatments: overviews versus intervention reviews. Abstracts of the 19th Cochrane Colloquium, 19-22 Oct 2011. Madrid, Spain: John Wiley & Sons; 2011. [Available online: http://www.cmim.cochrane.org/workshops-19th-cochrane-colloquium-october-2011. Follow link for workshop 1 slides. Accessed 30 Nov 2015.]
13. Becker L, Caldwell D, Higgins J, Li T, Salanti G, Schmid C. Comparing multiple interventions in Cochrane reviews. In: Comparing multiple interventions in Cochrane reviews. Cochrane Comparing Multiple Interventions Methods Group. 2013. http://www. cmim.cochrane.org/comparing-multiple-interventions-cochrane-reviews [Follow link saying "A background paper explaining the rationale"]. Accessed 30 Nov 2015.
14. Becker LA, Caldwell D, Salanti G, Li T. Editorial considerations for reviews that compare multiple interventions. In: Editorial considerations for reviews that compare multiple interventions. Cochrane Comparing Multiple Interventions Methods Group. 2013. http://www.cmimg.cochrane.org/editorial-considerations-reviews-compare-multiple-interventions [Follow links for slides]. Accessed 30 Nov 2015.
15. Becker LA, Li T, Caldwell D. Comparing multiple treatments 1: overview or intervention review. Abstracts of the 20th Cochrane Colloquium, 30 Sep-03 Oct 2012. Auckland, New Zealand: John Wiley & Sons; 2012. [Available online: http://www.cmim.cochrane.org/20th-cochrane-colloquium-auckland-2012. Follow link saying "Click here for slides from this workshop" for workshop 1 slides. Accessed 30 Nov 2015.]
16. Becker LA, Oxman AD. Chapter 22: overviews of reviews. In: Higgins JPT, Green S, editors. Cochrane handbook for systematic reviews of interventions (version 5.1.0). The Cochrane Collaboration; 2011.
17. Caldwell DM, Welton NJ, Ades AE. Mixed treatment comparison analysis provides internally coherent treatment effect estimates based on overviews of reviews and can reveal inconsistency. J Clin Epidemiol. 2010;63(8):875-82.
18. Li T. Comparing multiple treatments: − intervention review or overview − part 1. Abstracts of the 21st Cochrane Colloquium, 19-23 Sep 2013. Quebec City, Canada: John Wiley & Sons; 2013. [Available online: http://www.cmim.cochrane.org/quebec-2013. Follow link for workshop 1 slides. Accessed 30 Nov 2015.]
19. Li T, Becker LA. Comparing multiple treatments 1: intervention review or overview? Abstracts of the 22nd Cochrane Colloquium, 21-26 Sep 2014. Hyderabad, India: John Wiley & Sons; 2014.
20. Salanti G, Becker LA, Caldwell D, Higgins J, Li T, Schmid C. Evolution of Cochrane intervention reviews and overviews of reviews to better accommodate comparisons among multiple interventions. In: Evolution of Cochrane intervention reviews and overviews of reviews to better accommodate comparisons among multiple interventions. Cochrane Comparing Multiple Interventions Methods Group. 2011. http://www.cmim.cochrane.org/ Milan-report [Follow link saying "To download a copy of the full report in PDF format click here]. Accessed 30 Nov 2015.
21. Agency for Healthcare Research and Quality Evidence-based Practice Centre Program Working Group 3: integrating bodies of evidence: systematic reviews and individual studies. Interview transcript: Lorne Becker. 2014. [Unpublished.]

1. Cochrane Comparing Multiple Interventions Methods Group. Comparing Multiple Interventions Methods Group meeting minutes. In: Paris meeting - comparing multiple interventions in Cochrane reviews. Cochrane Comparing Multiple Interventions Methods Group. 2012. http://www.cmim.cochrane.org/Paris-2012 [Follow link saying "Minutes of the meeting"]. Accessed 30 Nov 2015.
2. Cochrane Comparing Multiple Interventions Methods Group. Editorial decision tree for overviews. In: Comparing multiple interventions in Cochrane reviews. Cochrane Comparing Multiple Interventions Methods Group. (2013). http://www.cmim.cochrane.org/comparing-multiple-interventions-cochrane-reviews [Follow link saying "An editorial decision tree"]. Accessed 30 Nov 2015.
3. Cochrane Comparing Multiple Interventions Methods Group. Methods innovation fund - stream 1. http://www.cmim.cochrane.org/methods-innovation-fund-stream-1 (2013). Accessed 30 Nov 2015.
4. Cochrane Comparing Multiple Interventions Methods Group. Multiple intervention reviews: reflections from CoEds discussions this week. In: Paris meeting - comparing multiple interventions in Cochrane reviews. Cochrane Comparing Multiple Interventions Methods Group. 2012. http://www.cmim.cochrane.org/Paris-2012 [Follow link saying "Powerpoint summary of CoEds discussion"]. Accessed 30 Nov 2015.
5. Cochrane Comparing Multiple Interventions Methods Group. Review type and methodological considerations - background paper for the first part of the Paris CMIMG discussion. In: Paris meeting - comparing multiple interventions in Cochrane reviews. Cochrane Comparing Multiple Interventions Methods Group. 2012. http://www.cmim. cochrane.org/Paris-2012 [Follow link saying "Background paper"]. Accessed 30 Nov 2015.
6. Worswick J, Wayne SC. Methodology of meta-synthesis: overviews of systematic reviews. Canadian Agency for Drugs and Technology in Health Symposium, 03-05 Apr 2011. Vancouver, Canada.
7. Baker PR, Costello JT, Dobbins M, Waters EB. The benefits and challenges of conducting an overview of systematic reviews in public health: a focus on physical activity. J Public Health (Oxf). 2014;36(3):517-21.
8. Cooper H, Koenka AC. The overview of reviews: unique challenges and opportunities when research syntheses are the principal elements of new integrative scholarship. Am Psychol. 2012;67(6):446-62.
9. Caird J, Sutcliffe K, Kwan I, Dickson K, Thomas J. Mediating policy-relevant evidence at speed: are systematic reviews of systematic reviews a useful approach? Evid Policy. 2015;11(1):81-97.
10. Thomas J. What should we expect from overviews? 23rd Cochrane Colloquium, Overviews of Systematic Reviews Post-Colloquium Symposium, 08 Oct 2015. Vienna, Austria.
11. Aromataris E, Fernandez R, Godfrey C, Holly C, Khalil H, Tungpunkom P. Methodology for JBI umbrella reviews. 2013. [Unpublished.]
12. Aromataris E, Fernandez R, Godfrey CM, Holly C, Khalil H, Tungpunkom P. Summarizing systematic reviews: methodological development, conduct and reporting of an umbrella review approach. Int J Evid Based Healthc. 2015;13(3):132-40.
13. Aromataris E, Fernandez R, Godfrey C, Holly C, Khalil H, Tungpunkom P, editors. The Joanna Briggs Institute reviewers' manual 2014: methodology for JBI umbrella reviews. University of Adelaide: Joanna Briggs Institute; 2014.
14. Joanna Briggs Institute Umbrella Review Methods Group. Umbrella review - systematic review methods group progress report. 2013. [Unpublished.]
15. Norwegian Knowledge Centre for the Health Services. 2: Vare ulike produkter [2: Our various products]. In: Slik oppsummerer vi forskning: handbok for Nasjonalt kunnskapssenter for helsetjenesten (reviderte utg 3.2) [How we summarize research: handbook for Norwegian Knowledge Centre for the Health Services (revised edition 3.2)]. Oslo: Norwegian Centre for the Health Services; 2013.
16. Smith V, Devane D, Begley CM, Clarke M. Methodology in conducting a systematic review of systematic reviews of healthcare interventions. BMC Med Res Methodol. 2011;11(1):15.
17. Hemming K, Bowater RJ, Lilford RJ. Pooling systematic reviews of systematic reviews: a Bayesian panoramic meta-analysis. Stat Med. 2012;31(3):201-16.
18. Conn VS, Coon Sells TG. WJNR welcomes umbrella reviews. West J Nurs Res. 2014;36(2):147-51.
19. Pieper D, Antoine SL, Mathes T, Neugebauer EA, Eikermann M. Systematic review finds overlapping reviews were not mentioned in every other overview. J Clin Epidemiol. 2014;67(4):368-75.
20. Pieper D, Antoine SL, Neugebauer EA, Eikermann M. Up-to-dateness of reviews is often neglected in overviews: a systematic review. J Clin Epidemiol. 2014;67(4):1302-8.

**Documents that describe an author team's experience conducting one or more published overviews (11 documents produced by 9 research groups)**

1. Foisy M, Becker LA, Chalmers JR, Boyle RJ, Simpson EL, Williams HC. Mixing with the ‘unclean’: including non-Cochrane reviews alongside Cochrane reviews in overviews of reviews. Abstracts of the 19th Cochrane Colloquium, 19-22 Oct 2011. Madrid, Spain: John Wiley & Sons; 2011.
2. Thomson D, Foisy M, Oleszczuk M, Wingert A, Chisholm A, Hartling L. Overview of reviews in child health: evidence synthesis and the knowledge base for a specific population. Evid Based Child Health. 2013;8(1):3-10.
3. Ryan RE, Kaufman CA, Hill SJ. Building blocks for meta-synthesis: data integration tables for summarising, mapping, and synthesising evidence on interventions for communicating with health consumers. BMC Med Res Methodol. 2009;9:16.
4. Flodgren GS, Shepperd S, Eccles, M. Challenges facing reviewers preparing overviews of reviews. Abstracts of the 19th Cochrane Colloquium, 19-22 Oct 2011. Madrid, Spain: John Wiley & Sons; 2011.
5. Pantoja T, Opiyo N, Ciaponni A, Herrera C, Lewin S, Oxman A, et al. Strategies for improving health systems in low-income countries: lessons learnt from four overviews of systematic reviews of health systems interventions. Abstracts of the 23rd Cochrane Colloquium, 03-07 Oct 2015. Vienna, Austria: John Wiley & Sons; 2015.
6. Tanjong Ghogomu E, Maxwell L, Singh J, Christensen R, Wells G, Buchbinder R, et al. Overcoming methodological challenges associated with network meta-analysis: the experience of the Musculoskeletal Group. Abstracts of the 19th Cochrane Colloquium, 19-22 Oct 2011. Madrid, Spain: John Wiley & Sons; 2011.
7. Pollock A, Farmer SE, Brady MC, Langhorne P, Mead GE, Mehrholz J, et al. Completing the first Cochrane overview of stroke reviews: experiences of the Cochrane Stroke Group. Abstracts of the 23rd Cochrane Colloquium, 03-07 Oct 2015. Vienna, Austria: John Wiley & Sons; 2015.
8. Kramer SFL, Langendam M, Elbers R, Scholten R, Hooft L. Preparing an overview of reviews: lessons learned. Abstracts of the 17th Cochrane Colloquium, 11-14 Oct 2009. Singapore, Singapore: John Wiley & Sons; 2009.
9. Piso B, Semlitsch T, Reinsperger I, Breuer J, Kaminski-Hartenthaler A, Kien C, et al. Praxiserfahrungen mit overviews of reviews - wertvolle entscheidungsunterstützung oder wissenschaftliche fingerübung? [Practical experience with overviews of reviews - valuable decision aid or academic exercise?]. Z Evid Fortbild Qual Gesundhwes. 2015;109(4-5):300-8.
10. Rojas ML, Lozano J, Sola I, Bonfill X. Incorporating the GRADE approach in overviews of systematic reviews: an example from an overview in neonatal respiratory care. Abstracts of the 19th Cochrane Colloquium, 19-22 Oct 2011. Madrid, Spain: John Wiley & Sons; 2011.
11. Elliott L, Crombie IK, Irvine L, Cantrell J, Taylor J. The effectiveness of public health nursing: the problems and solutions in carrying out a review of systematic reviews. J Adv Nurs. 2004;45(2):117-25.
